# Clinical and Inflammatory Correlates of Negative Symptom Dimensions in Schizophrenia: Cross-Sectional Evidence for Neutrophil-to-Lymphocyte Ratio Associations With Motivational Deficits in Hospitalized Patients

**DOI:** 10.7759/cureus.100390

**Published:** 2025-12-30

**Authors:** Cosmin-Ioan Moga, Octavia Capatina, Catalina Crisan, Mihaela Fadgyas Stanculete, Ioana Miclutia

**Affiliations:** 1 Neuroscience, Iuliu Hațieganu University of Medicine and Pharmacy, Cluj-Napoca, ROU; 2 Neuroscience/Psychosomatic Medicine, Iuliu Hatieganu University of Medicine and Pharmacy, Cluj-Napoca, ROU

**Keywords:** bmi, bnss, clozapine, complement c4, crp levels, hospital, inflammation, negative symptoms, neutrophil-to-lymphocyte ratio (nlr), schizophrenia

## Abstract

Background: This study examined negative symptom dimensions and their biological correlates in patients with schizophrenia under routine hospital conditions.

Objective: To assess whether demographic, clinical, and inflammatory markers, that is, C-reactive protein (CRP), complement component 4 (C4), and the neutrophil-to-lymphocyte ratio (NLR), contribute to variation across negative symptom domains measured using two complementary instruments.

Methodology: A cross-sectional study was conducted at the Clinics I-II of Psychiatry, Cluj-Napoca, Romania (May-September 2023). Forty-five inpatients with schizophrenia were evaluated using the Brief Negative Symptom Scale (BNSS) and the Positive and Negative Syndrome Scale (PANSS). Fasting CRP and C4 were measured, and NLR was calculated from differential blood counts. Analyses included descriptive statistics, Shapiro-Wilk testing, parametric/nonparametric comparisons, and Pearson/Spearman correlations. Multiple linear regressions included biomarkers as predictors and were adjusted for sex, age, body mass index (BMI), smoking, chlorpromazine-equivalent dose, and clozapine treatment; models were compared using Akaike Information Criterion (AIC) (ΔAIC ≤ 2). Sensitivity analyses excluded clozapine-treated patients and additionally adjusted for PANSS Positive and General Psychopathology totals.

Results: BMI correlated negatively with negative symptoms (PANSS negative: ρ = −.43) and positively with CRP (ρ = .67). Stratified analyses revealed a CRP-Avolition association in non-overweight patients (ρ = .49) and stronger neutrophil-to-lymphocyte ratio (NLR)-motivational domain correlations in clozapine-treated patients (Asociality/Motivation and Pleasure (MAP): ρ = .59-.66). In covariate-adjusted, AIC-selected models, NLR remained significant for Asociality, PANSS Emotional Withdrawal, and MAP (adjusted R² = .19-.24; partial R² = .10-.16). CRP was retained only for Avolition with limited explained variance (adjusted R² = .03; partial R² = .09). C4 did not enter any final model.

Conclusion: In this inpatient cohort, NLR was the most consistent correlate of motivational negative symptoms, while CRP showed a weak association with Avolition, and C4 showed no clear relationship. Findings are hypothesis-generating and require replication in larger, longitudinal samples.

## Introduction

Schizophrenia is a debilitating psychiatric disorder that affects approximately 0.7% of the global population [1]. It is associated with a two-to-threefold increase in mortality risk compared to the general population [1] and accounts for more than 50% of inpatient admissions among all psychiatric diagnoses [2]. Historically, the schizophrenia phenotype has been conceptualized as polarized between positive symptoms, such as hallucinations and delusions, that add to normal mental and behavioral functioning, and negative symptoms, which indicate a loss or reduction in normal cognitive, emotional, and behavioral abilities [3]. Although positive symptoms often precipitate hospitalization, negative symptoms are the strongest predictors of poor functional outcomes and low remission rates [4]. The current understanding of negative symptoms, based on the National Institute of Mental Health-Measurement and Treatment Research to Improve Cognition in Schizophrenia (NIMH-MATRICS) Consensus, highlights five key domains: Anhedonia, Asociality, Avolition, Blunted Affect, and Alogia [5]. These are typically categorized into two main factors: the Motivational Deficit domain and the Expressive Deficit domain [6]. In line with this framework, the Brief Negative Symptom Scale (BNSS) was developed to enhance the sensitivity and specificity of negative symptom assessment compared to earlier, broader instruments, such as the Positive and Negative Syndrome Scale (PANSS) [7].

Over the past three decades, converging evidence has highlighted a proinflammatory signature in schizophrenia, marked by persistent immune alterations across both central and peripheral systems [8]. Neuroimaging studies indicate microglial activation, while biofluid analyses consistently report elevated inflammatory markers in serum, plasma, and cerebrospinal fluid [8]. These abnormalities include dysregulation of cytokines, such as IL-1, IL-6, and TNF-α [9], elevated C-reactive protein [10], and altered complement system activity, most notably involving complement component C4 [11]. Structural variation within the C4 locus, particularly alleles that enhance C4A expression, remains one of the strongest genetic risk signals for schizophrenia and is thought to contribute to excessive complement-mediated synaptic pruning [12]. Peripheral immune-cell alterations, such as elevated neutrophil and monocyte counts, further support sustained immune dysregulation in the disorder [13]. Nevertheless, emerging evidence suggests that immune abnormalities may be present only in a subset of patients [8], and recent studies increasingly link the negative symptom phenotype to this distinct immunological profile [14,15].

We hypothesized that the enhanced sensitivity of the BNSS, when used alongside the PANSS, would allow a more refined characterization of clinico-biological profiles of negative symptoms in schizophrenia. Accordingly, in this cross-sectional study of hospitalized patients with schizophrenia, our aims were: (1) to examine how negative symptoms relate to key sociodemographic and clinical factors; (2) to investigate the relationships between negative symptoms and routinely available peripheral inflammatory markers, that is, C-reactive protein (CRP), neutrophil-to-lymphocyte ratio (NLR), and complement C4, and whether these relationships vary across clinically relevant subgroups; and (3) to assess the combined contributions of clinical and biological factors to negative symptom dimensions.

## Materials and methods

Study design and participants

This cross-sectional observational study was conducted between May and September 2023 in the open inpatient units of Psychiatry Clinics I and II, Cluj-Napoca, Romania. Participants were consecutively recruited from these wards.

Inclusion criteria required (1) a DSM-5 diagnosis [16] of schizophrenia confirmed via a structured clinical interview specific to the admitting units, (2) age between 18 and 65 years, and (3) clinical stability, which is adequate for standardized psychometric assessment and marked by the absence of acute exacerbation or medication adjustment in the past two weeks.

Exclusion criteria included the (1) presence of acute infections or recent traumatic injuries; (2) a history of autoimmune or chronic infectious diseases; (3) elevated C-reactive protein (CRP ≥ 10 mg/L); (4) hematological abnormalities, including clozapine-associated neutropenia (neutrophils < 1500/mm³); (5) intellectual disability (IQ < 70); and (6) comorbid psychiatric disorders or current substance use disorders.

Data collection and instruments

Data were organized into sociodemographic and clinical, psychometric, and biological domains and entered into a dedicated Microsoft Excel Version 16.92 (Microsoft Corp., Redmond, WA, USA) database. Sociodemographic information was collected through structured interview forms, psychiatric symptoms were evaluated using standardized psychometric scales, and biological data were obtained from laboratory assays performed under routine clinical conditions.

Sociodemographic and clinical data included sex, age, age at illness onset, illness duration (years), family psychiatric history (coded as yes/no), education level (up to four years of schooling; secondary: five to 12 years; university level: university or equivalent), living environment (urban/rural), civil status (single/partnered), social status (employed/unemployed, the latter including both individuals without an occupation or on medical disability), body mass index (BMI), and smoking intensity (cigarettes per day). BMI was categorized as non-overweight (<25) or overweight (≥25). Antipsychotic doses were standardized to daily chlorpromazine (CPZ) equivalents (mg/day).

Psychometric data were obtained using the BNSS [7] and the PANSS [17]. The BNSS comprises 13 items rated on a 0-6 Likert scale, covering the five core negative symptom domains plus lack of normal distress, yielding a total score range of 0-78. For this study, we used the Romanian version of the BNSS with permission from the original authors (Kirkpatrick et al. [7]) for non-commercial academic use. This version was validated in a prior study of Romanian-speaking patients with schizophrenia and demonstrated excellent internal consistency (Cronbach’s α = 0.94) and good convergent and discriminant validity [18]. In line with validated factor models, BNSS items were further grouped into two higher-order dimensions: the Motivational Deficit (MAP) domain (Anhedonia, Asociality, Avolition) and the Expressive Deficit (EXP) domain (Blunted Affect, Alogia). The PANSS consists of 30 items rated from 1 (absent) to 7 (extreme), organized into three subscales: Positive (7 items), Negative (7 items), and General Psychopathology (16 items). In the present study, PANSS negative symptoms were evaluated using the Negative Symptom Factor Score [4], which includes items N1 (Blunted Affect), N2 (Emotional Withdrawal), N3 (Poor Rapport), N4 (Passive Social Withdrawal), and N6 (Lack of Spontaneity), alongside the PANSS Negative subscale total score. Additionally, PANSS Positive and General Psychopathology subscale total scores were also analyzed for comparison purposes. A validated Romanian version of the PANSS was used, demonstrating good psychometric properties (Cronbach’s α: Positive = 0.83, Negative = 0.71, General Psychopathology = 0.74) [17], and was officially licensed for this study (license ID: MN-00014995).

Biological data included fasting serum levels of CRP, complement component C4, and the NLR. Fasting venous blood was collected before 8:00 a.m. Serum for CRP and C4 determination was obtained in serum separator tubes, while hematology samples were collected in ethylenediaminetetraacetic acid (EDTA)-coated tubes. All samples were transported to the Central Laboratory of the Cluj County Emergency Clinical Hospital and processed according to standardized clinical laboratory procedures.

Biomarker assessment

CRP concentrations were measured using a latex-enhanced immunoturbidimetric assay on a Beckman Coulter AU analyzer (CRP Latex OSR6199/OSR6299) (Beckman Coulter, Inc., Brea, CA, USA). The assay supports both standard and highly sensitive configurations, with a lower analytical range extending to 0.08 mg/L and an estimated limit of detection of 0.02-0.07 mg/L. Intra- and inter-assay coefficients of variation were <6% at low CRP concentrations, and calibration was traceable to the IFCC international reference material (CRM 470). The laboratory reference interval for CRP was <5 mg/L, with values <1 mg/L reflecting low-grade systemic inflammation. 

Serum C4 was quantified using an immunoturbidimetric assay on a Beckman Coulter AU analyzer (C4 OSR6160) with goat anti-human C4 antibodies. The assay was linear over 0.08-1.50 g/L, with a limit of detection of approximately 0.002 g/L and intra-/inter-assay coefficients of variation ≤2.5%. Calibration was traceable to the ERM®-DA470 reference standard, and the laboratory reference interval was 0.10-0.40 g/L. Given that C4 behaves as an acute-phase reactant, values were interpreted as reflecting state-dependent immune activation. 

Complete blood count parameters were measured using automated hematology analyzers under routine clinical conditions. The NLR was calculated as the absolute neutrophil count divided by the absolute lymphocyte count.

Statistical analysis

All analyses were conducted in R (version 4.4.2; R Foundation for Statistical Computing, Vienna, Austria) using dplyr, tidyr, and purrr (Posit Software, PBC, Boston, MA, USA) for data management; stats (R Foundation for Statistical Computing), broom, car, sandwich, and imtest (independently developed open-source software, distributed via Comprehensive R Archive Network) for regression modeling and diagnostics; and ggplot2 (Posit Software) for visualization. Categorical variables were reported as frequencies and percentages, while continuous variables were reported as means and standard deviations. Normality was assessed using the Shapiro-Wilk test and visual inspection of residuals. Between-group comparisons used Student’s t-tests or Wilcoxon tests, as appropriate. All tests were two-tailed with p < .05. Exploratory bivariate associations between negative symptom dimensions, inflammatory biomarkers, and clinical variables were examined using Pearson or Spearman correlations in the full sample and stratified by relevant clinical variables. Given the modest sample size and exploratory nature of the study, no formal correction for multiple testing was applied; findings are interpreted as hypothesis-generating. 

Negative symptom domains showing notable associations in preliminary analyses were entered into multivariable regression models. CRP, NLR, and C4 served as primary biomarker predictors, while sex, age, BMI, smoking, chlorpromazine (CPZ)-equivalent dose, and clozapine treatment were included as covariates. For each outcome, a structured family of linear regression models was fitted, comprising: (1) covariates only; (2) covariates plus individual biomarkers; (3) models including combinations of biomarkers; and (4) models incorporating biologically and clinically plausible interaction terms. Model selection was based on Akaike’s Information Criterion (AIC), retaining the most parsimonious model within ΔAIC ≤ 2. Model assumptions were evaluated using Q-Q and scale-location plots; multicollinearity was assessed with variance inflation factors; and HC3 robust standard errors were used. Partial R² quantified biomarker-specific variance, and 10-fold cross-validation estimated out-of-sample RMSE and R². Sensitivity analyses included restriction to non-clozapine-treated patients and additional adjustment for PANSS Positive and PANSS General scores. All results are interpreted cautiously as hypothesis-generating.

## Results

Sample description

The study included 45 adults with schizophrenia. Most participants were female individuals (60%), unpartnered (73%), and lived in urban areas (82%). The mean age was 42.80 years (SD = 12.28), and the mean illness duration was 17.16 years (SD = 10.83). Mean symptom severity scores were 93.38 (SD = 18.41) for the PANSS Total, 26.96 (SD = 7.12) for the PANSS Negative subscale, and 38.84 (SD = 13.27) for the BNSS Total. Mean inflammatory marker values were 0.75 mg/L (SD = 0.69) for CRP, 0.34 g/L (SD = 0.06) for C4, and 2.83 (SD = 1.47) for the NLR. The mean BMI was 28.48 (SD = 8.14). The mean CPZ-equivalent dose was 509.45 mg/day (SD = 272.94). Clozapine was the most frequently prescribed antipsychotic (28.89%), while amisulpride and aripiprazole were the least frequently prescribed (4.44% each). These data are presented in Table 1.

**Table 1:** Characteristics of Study Sample. SD = Standard Deviation; BMI = Body Mass Index; PANSS = Positive and Negative Symptoms Scale [17]; BNSS = Brief Negative Symptom Scale [7]; CRP = C-reactive protein; C4 = Complement C4; NLR = Neutrophil-to-Lymphocyte Ratio; nn refers to the non-normal distributed values according to the Shapiro-Wilk test.

Variable	Value/Level	n	%	Mean (*SD*)
Sex	Female	27	60.00	-
Education Level	Secondary	29	64.44	-
Civil Status	Single	33	73.33	-
Social Status	Unemployed	39	86.67	-
Family History	Yes	12	26.67	-
Living Environment	Urban	37	82.22	-
Overweight	Yes	26	57.78	-
Age (years)	-	45	-	42.80 (12.28)
Age of onset (years)^nn^	-	45	-	25.81 (7.19)
Duration of illness (years)^nn^	-	45	-	17.16 (10.83)
BMI (kg/m^2^)^nn^	-	45	-	28.48 (8.14)
Smoking (cigarettes per day)^nn^	-	45	-	9.13 (11.92)
PANSS Total	-	45	-	93.38 (17.80)
PANSS Positive	-	45	-	22.27 (5.99)
PANSS Negative	-	45	-	26.96 (8.20)
PANSS General	-	45	-	44.16 (9.06)
BNSS Total^nn^	-	45	-	38.84 (17.47)
Anhedonia	-	45	-	8.62 (4.51)
Asociality	-	45	-	6.29 (2.90)
Avolition^nn^	-	45	-	6.24 (2.65)
Blunted Affect^nn^	-	45	-	10.04 (5.76)
Alogia^nn^	-	45	-	5.00 (3.98)
Lack of Normal Distress^nn^	-	45	-	2.64 (1.38)
MAP	-	45	-	21.16 (8.91)
EXP^nn^	-	45	-	15.04 (8.99)
CRP (mg/L)^nn^	-	45	-	0.75 (0.96)
C4 (g/L)	-	43	-	0.34 (0.10)
NLR^nn^	-	45	-	2.83 (1.41)
Chlorpromazine equivalent doses (mg/day)^nn^	-	45	-	509.45 (272.94)

Negative symptoms and their associations with sociodemographic and clinical variables

Sociodemographic Associations

Group-based analyses were conducted to assess differences in negative symptom severity across clinical and sociodemographic factors. Overall, no significant differences were observed for sociodemographic variables across core negative symptom domains, with two exceptions: men scored higher than women on the BNSS Lack of Normal Distress item (W = 140.00, p = .014, 95% CI (0.01, 2.00)), and single participants showed greater passive social withdrawal (N4) than partnered participants (W = 118.00, p = .037, 95% CI (0.01, 2.00)).

Clinical Associations

BMI showed the most consistent associations with negative symptoms, such that higher BMI was related to lower symptom severity (PANSS Negative: ρ(43) = −.43, p = .003), with the strongest effects observed for Poor Rapport (N3: ρ(43) = −.51, p < .001) and the EXP factor (ρ(43) = −.45, p = .002; see Table 2). Consistent with these dimensional findings, participants in the overweight group exhibited significantly lower negative symptom severity than non-overweight participants, with reduced scores on Blunted Affect (N1; W = 333.00, p = .046, 95% CI (0.01, 2.00)), Poor Rapport (N3; W = 359.00, p = .009, 95% CI (0.01, 2.00)), and the PANSS Negative total score, t(41.1) = 2.09, p = .043, 95% CI (0.16, 9.65). The antipsychotic class was not consistently associated with symptom severity, although patients treated with clozapine tended to have higher negative symptom scores (e.g., PANSS Negative: t(24.1) = -2.53, p = .019, 95% CI (-11.32, -1.14); Table 3). Patients without a family history of psychosis scored higher on Poor Rapport (N3; W = 285.00, p = .024, 95% CI (0.01, 2.00)) and Lack of Spontaneity (N6; W = 276.00, p = .044, 95% CI (0.01, 3.00)) than those with a positive family history. Hospitalization history was not significantly related to symptom severity, though non-significant trends were observed for Anhedonia and Alogia (all ps = .07-.08).

**Table 2:** Correlations Between Negative Symptoms and Clinical Variables. Negative symptoms were assessed using two instruments: Positive and Negative Syndrome Scale (PANSS) [17] items N1, N2, N3, N4, N6, and the PANSS Negative Subscale; and the Brief Negative Symptom Scale (BNSS) [7], which provided scores for total severity, Anhedonia, Asociality, Avolition, Blunted Affect, Alogia, as well as the MAP (Motivation and Pleasure) and EXP (Expressive) factor. The values represent Pearson (r) or Spearman (ρ) correlation coefficients; bold = statistical significance: * = p < 0.05; ** = p < 0.01; *** = p < 0.001; CPZ eq. = chlorpromazine-equivalent dose (mg/day).

Symptom	Age	Onset	Duration	BMI	Smoking	CPZ eq.
Blunted Affect (N1)	-0.09	-0.04	-0.05	-0.43**	-0.02	-0.01
Emotional Withdrawal (N2)	0.07	-0.04	0.11	-0.29	0.00	-0.06
Poor Rapport (N3)	-0.08	-0.14	-0.01	-0.50***	-0.03	-0.07
Passive Social Withdrawal (N4)	0.00	-0.23	0.14	-0.32*	0.08	-0.21
Lack of Spontaneity (N6)	-0.06	-0.12	-0.03	-0.34*	-0.08	-0.13
PANSS Negative	-0.01	-0.13	0.09	-0.43**	-0.02	-0.09
BNSS Total	-0.01	-0.10	0.03	-0.40**	-0.09	-0.20
Anhedonia	0.11	0.05	0.06	-0.32*	-0.21	-0.30*
Lack of Normal Distress	-0.04	-0.11	0.02	-0.42**	0.02	-0.27
Asociality	0.05	-0.16	0.14	-0.11	-0.05	-0.13
Avolition	-0.01	-0.12	0.03	-0.42**	-0.06	-0.10
Blunted Affect	-0.16	-0.17	-0.10	-0.42**	-0.06	-0.24
Alogia	0.05	-0.04	0.09	-0.31*	-0.12	-0.29
MAP	-0.09	-0.13	-0.04	-0.45**	-0.06	-0.11
EXP	-0.08	-0.15	-0.01	-0.17	0.19	-0.06
PANSS Positive	-0.14	-0.15	-0.03	-0.05	0.24	0.24
PANSS General	-0.08	-0.10	-0.01	-0.31*	-0.12	-0.21

**Table 3:** Group Comparisons on Negative Symptoms by Overweight Status and Clozapine Treatment. Negative symptoms were assessed using two instruments: Positive and Negative Syndrome Scale (PANSS) [17] items N1, N2, N3, N4, N6, and the PANSS Negative Subscale; and the Brief Negative Symptom Scale (BNSS) [7], which provided scores for total severity, Anhedonia, Asociality, Avolition, Blunted Affect, Alogia, as well as the MAP (Motivation and Pleasure) and EXP (Expressive) factor. The values are group means. t = t-test statistic; W = Wilcoxon test statistic (depending on normality). Bold indicates p < .05. Confidence intervals are 95%.

Symptom	Test (Overweight)	Not Overweight	Overweight	95% CI	Test (Clozapine)	Non-Clozapine	Clozapine	95% CI
Blunted Affect (N1)	W = 333.00, p = 0.046	4.63	3.50	(0.01, 2.00)	W = 139.00, p = 0.082	3.66	4.77	(-2.00, 0.01)
Emotional Withdrawal (N2)	t = 1.22, p = 0.231	4.16	3.62	(-0.36, 1.44)	W = 118.50, p = 0.022	3.53	4.62	(-2.00, -0.01)
Poor Rapport (N3)	W = 359.00, p = 0.009	4.37	3.12	(0.01, 2.00)	W = 108.50, p = 0.012	3.25	4.62	(-3.00, -0.01)
Passive Social Withdrawal (N4)	W = 293.00, p = 0.285	4.53	4.04	(-0.01, 1.00)	W = 134.00, p = 0.060	3.97	4.92	(-2.00, 0.01)
Lack of Spontaneity (N6)	W = 282.00, p = 0.420	3.79	3.38	(-1.00, 1.00)	W = 124.00, p = 0.033	3.19	4.46	(-3.00, -0.01)
PANSS Negative	t = 2.09, p = 0.043	29.79	24.88	(0.16, 9.65)	t = -2.53, p = 0.019	25.16	31.38	(-11.32, -1.14)
BNSS Total	t = 1.63, p = 0.110	43.58	35.38	(-1.92, 18.31)	t = -1.72, p = 0.096	36.22	45.31	(-19.92, 1.74)
Anhedonia	t = 0.84, p = 0.407	9.26	8.15	(-1.56, 3.78)	t = -1.67, p = 0.106	7.97	10.23	(-5.04, 0.52)
Asociality	t = 1.94, p = 0.059	7.21	5.62	(-0.06, 3.25)	t = -2.04, p = 0.055	5.72	7.69	(-3.99, 0.04)
Avolition	t = 0.27, p = 0.791	6.37	6.15	(-1.41, 1.84)	W = 184.50, p = 0.562	6.06	6.69	(-2.00, 1.00)
Blunted Affect	W = 326.50, p = 0.068	11.89	8.69	(-0.01, 7.00)	W = 181.50, p = 0.513	9.72	10.85	(-4.00, 3.00)
Alogia	W = 304.50, p = 0.185	5.95	4.31	(-1.00, 4.00)	W = 135.00, p = 0.066	4.25	6.85	(-6.00, 0.01)
MAP	t = 1.12, p = 0.269	22.84	19.92	(-2.34, 8.18)	W = 138.50, p = 0.083	19.75	24.62	(-11.00, 1.00)
EXP	W = 320.50, p = 0.093	17.84	13.00	(-1.00, 11.00)	W = 161.50, p = 0.249	13.97	17.69	(-10.00, 2.00)
Lack of Normal Distress	t = 1.02, p = 0.316	2.89	2.46	(-0.43, 1.30)	W = 155.50, p = 0.182	2.50	3.00	(-1.00, 0.01)

Correlation analysis between negative symptoms and biomarkers

**Figure 1:**
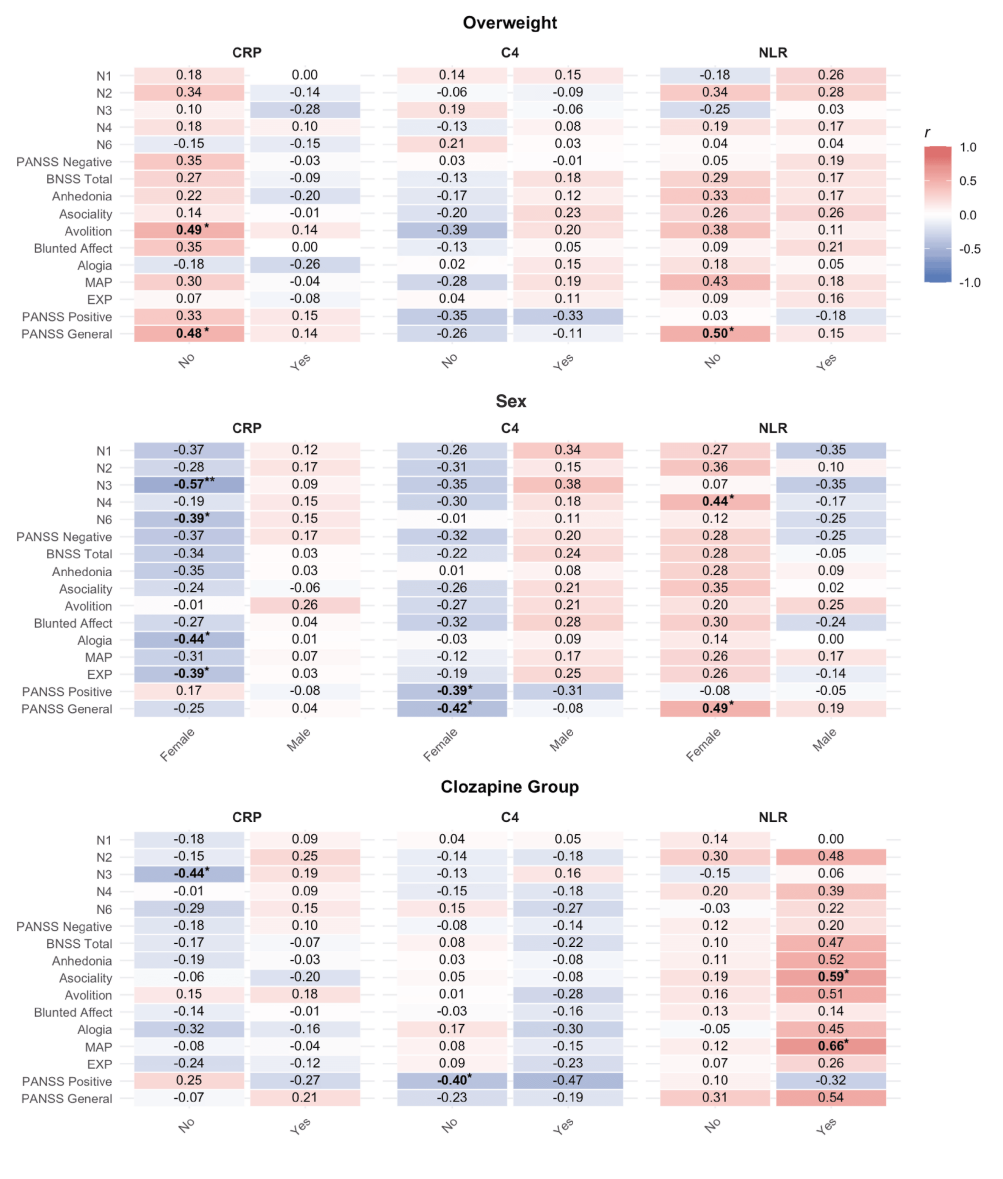
Stratified Correlations Between Negative Symptoms and Biomarkers by Overweight Status, Sex, and Clozapine Treatment Status. This heatmap shows Spearman and Pearson correlation coefficients within each cell between the three inflammatory biomarkers—C-reactive protein (CRP), complement component 4 (C4), and neutrophil-to-lymphocyte ratio (NLR)—and individual negative symptoms. Negative symptoms were assessed using two instruments: Positive and Negative Syndrome Scale (PANSS) [17] items N1, N2, N3, N4, N6, and the PANSS Negative Subscale; and the Brief Negative Symptom Scale (BNSS) [7], which provided scores for total severity, Anhedonia, Asociality, Avolition, Blunted Affect, Alogia, as well as the MAP (Motivation and Pleasure) and EXP (Expressive) factor. Correlations are grouped by overweight status (No = BMI < 25; Yes = BMI ≥ 25),  sex (female, male), and clozapine treatment status (Yes, No). Warmer colors (red) indicate stronger positive correlations, while cooler colors (blue) show negative correlations. Bold values with an asterisk above signify statistically significant correlations (*p < .05, **p < .01). This visualization emphasizes subgroup differences in biomarker–symptom relationships and highlights opposing trends between categories (see male-female for C4).

Bivariate associations between individual negative symptom dimensions and biomarkers were examined using Pearson and Spearman correlation analyses (see Appendix 1). PANSS Positive and General Psychopathology scores were included for comparative purposes. Before these analyses, potential associations between clinical covariates (age, age of onset, illness duration, BMI, smoking, CPZ-equivalent dosage, and clozapine status) and inflammatory markers were assessed. Among these, only CRP showed a significant correlation with BMI, ρ(43) = .67, p < .001, suggesting that BMI may confound the relationship between CRP and negative symptom severity. Mean NLR did not differ between clozapine-treated and non-clozapine patients (M = 2.87 vs. 2.74); W = 228, p = .39, 95% CI (−0.48, 1.10). Subsequent analyses between negative symptoms and biomarkers were stratified by sex, overweight status, and clozapine treatment (Figure 1).

C-reactive Protein

In the full sample, CRP showed mainly negative, though mostly non-significant, correlations with negative symptoms. When stratified by overweight status, CRP was positively associated with most negative symptoms in the non-overweight subgroup, with Avolition showing a significant correlation (ρ(17) = .49, p = .032). Stratification by clozapine status revealed a significant negative correlation between CRP and Poor Rapport (N3) only in those not treated with clozapine (ρ(30) = −.44, p = .012). CRP was unrelated to PANSS Positive scores but showed a positive association with the PANSS General Psychopathology subscale. Because CRP correlated strongly with BMI (ρ(43) = .67, p < .001), these results suggest that negative CRP-symptom associations in the full sample were confounded by BMI, whose inverse relationship with negative symptoms and positive relationship with CRP likely masked the direction of these associations. 

Complement Component 4

In the overall sample, C4 showed generally negative but non-significant correlations with negative symptom dimensions. Stratified analyses indicated a sex-related pattern: correlations in males were mostly positive, whereas those in females were consistently negative. This divergence was observed primarily for negative symptoms and was not evident for other symptom domains. C4 was also significantly negatively correlated with PANSS Positive and General Psychopathology scores in the full sample and among females. Partial correlations controlling for clinical covariates did not yield any significant associations.

Neutrophil-to-Lymphocyte Ratio

In the total sample, NLR did not show significant correlations with negative symptoms. However, sex stratification revealed a significant positive correlation between NLR and Passive Social Withdrawal (N4) in females (ρ(25) = .44, p = .023). This pattern reflected a subtle inter-sex divergence, with a positive association in females and a non-significant negative trend in males--an inversion compared to the patterns observed with the other two biomarkers. Further stratification by clozapine treatment showed significant positive associations between NLR and both asociality (ρ(11) = .59, p = .034) and the MAP factor score (ρ(11) = .66, p = .013), but only in clozapine-treated individuals (see Figure 1). Among non-negative symptom domains, NLR was significantly correlated only with PANSS General Psychopathology scores.

Multivariable regression models examining associations between negative symptoms, biomarkers, and clinical covariates

**Figure 2:**
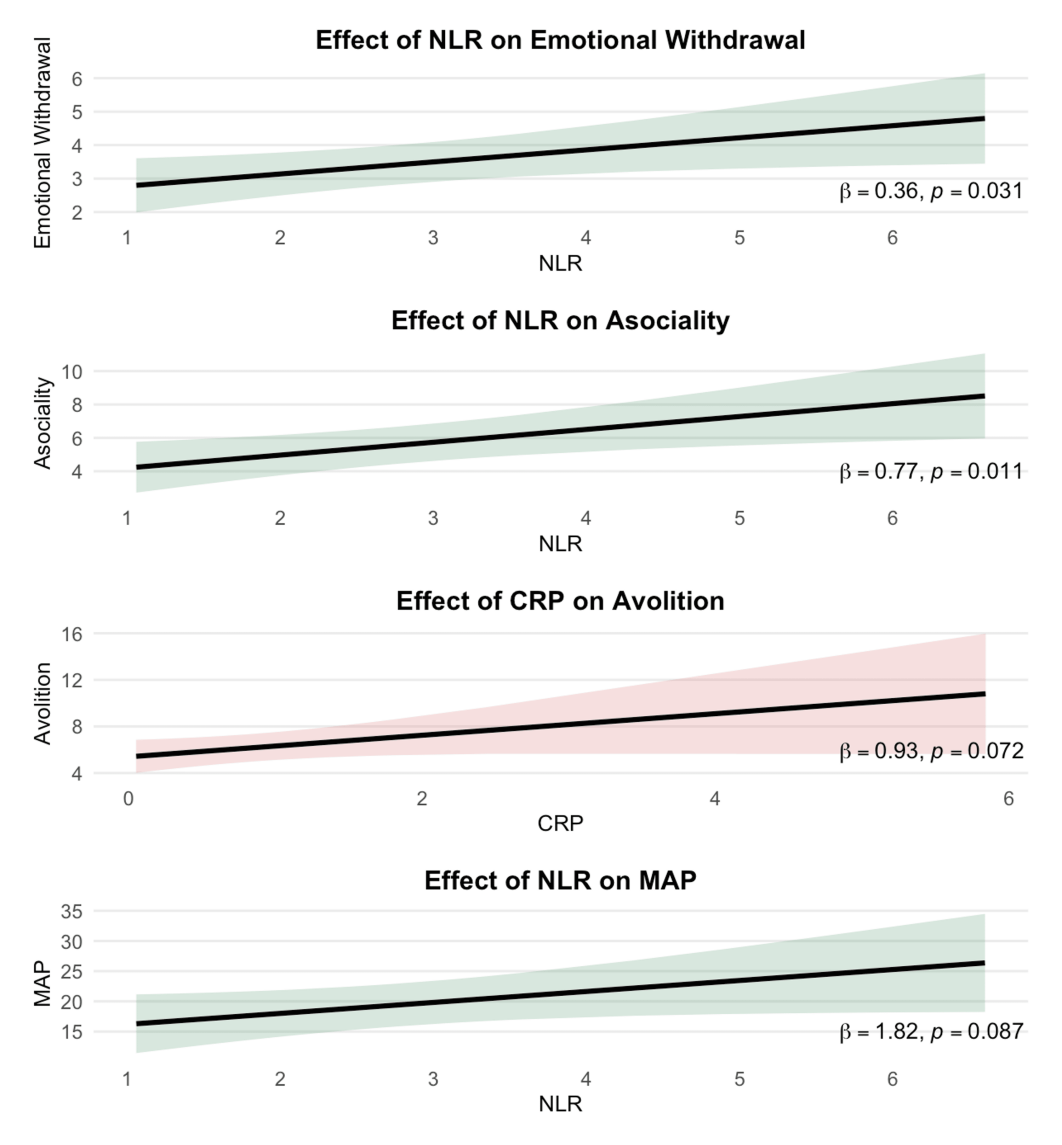
Adjusted Partial Effects of CRP and NLR on Motivational Negative Symptoms. Model-estimated negative symptom scores as a function of each focal biomarker, with 95% confidence ribbons. Associations are marginal (averaged over the empirical distributions of covariates). All models adjust for sex, age, BMI, smoking, chlorpromazine equivalents, and clozapine treatment, and use HC3-robust inference. Panels show: (1) the association between NLR and Emotional Withdrawal (PANSS [17] N2), (2) NLR and Asociality (BNSS [7]), (3) CRP and Avolition (BNSS [7]) from the model that also included NLR, and (4) NLR and the BNSS [7] Motivation and Pleasure (MAP) factor. Each panel reports the HC3-robust coefficient (*β*) and *p*-value for the focal biomarker. Wider confidence ribbons at biomarker extremes reflect lower data density. Overall, higher NLR was associated with more severe motivational negative symptoms (N2, Asociality, MAP), while CRP showed only a smaller, trend-level positive association with Avolition. CRP = C-reactive Protein; NLR = Neutrophil-to-Lymphocyte Ratio; BNSS = Brief Negative Symptom Scale; PANSS = Positive and Negative Syndrome Scale.

Model diagnostics supported the use of linear regression: residual Q-Q and scale-location plots revealed no substantial violations of normality or homoscedasticity (see Appendix 2), and all models were estimated with HC3 robust standard errors. Multicollinearity was low (all VIFs < 1.35). For each outcome, candidate models were ranked using AIC, with the most parsimonious model within a ΔAIC ≤ 2 retained. Interaction terms (CRP × BMI, NLR × clozapine, C4 × sex) did not improve model fit and were therefore not selected. For Asociality, the optimal model included NLR as a significant positive correlate (β = 0.77, p = .011, partial R² = .16; adjusted R² = .24, AIC = 212.10). NLR was also associated with Emotional Withdrawal (N2; β = 0.36, p = .031, partial R² = .13; adjusted R² = .20, AIC = 157.68), with additional effects of BMI (BMI; β = −0.06, p = .024) and clozapine use (β = 1.11, p = .032). The same covariate + NLR model was selected for the MAP score (β = 1.82, p = .087, partial R² = .10; adjusted R² = .19, AIC = 311.88), with trends for BMI (β = −0.32, p = .082) and clozapine (β = 5.18, p = .091). Avolition was best modeled with CRP as the sole biomarker (β = 0.93, p = .072, partial R² = .09; adjusted R² = .03, AIC = 216.29). Full model details are presented in Table 4 and visualized in Figure 2. Ten-fold cross-validation indicated limited out-of-sample explanatory performance across all models, with mean cross-validated R² values near zero or negative and relatively large RMSE values, indicating substantial optimism in in-sample model fit and a high risk of overfitting.

**Table 4:** Multivariable Models Linking Inflammatory Markers and Clinical Factors to Motivational Negative Symptoms. Negative symptoms were assessed using two instruments: Positive and Negative Syndrome Scale (PANSS) [17] item N2; and the Brief Negative Symptom Scale (BNSS) [7], which provided scores for Asociality, Avolition, and MAP (Motivation and Pleasure) factor. Models shown represent the final specifications selected using Akaike’s Information Criterion (AIC); when competing models fell within ΔAIC ≤ 2, the most parsimonious was retained. The F p-value reflects the HC3-robust omnibus test of overall model significance. Beta values are unstandardized regression coefficients; Std. Error (HC3) denotes heteroskedasticity-consistent standard errors; t and p values are based on HC3-robust inference. Partial R² (drop-one) is reported for continuous predictors and reflects the proportion of unique variance explained by each predictor conditional on all others. R² and adjusted R² summarize the variance explained by the full model. AIC values allow relative comparison of model fit within each outcome (lower AIC indicates better fit). Predictor coding: Gender: Male = male vs. female (reference); Clozapine: Yes = yes vs. no (reference). CPZ = chlorpromazine-equivalent daily antipsychotic dose. Continuous covariates are expressed in native units: Age (years), BMI (kg/m²), Smoking (cigarettes/day), CPZ (mg/day), NLR, and CRP (mg/L). All models use complete-case data (n = 43) and include only additive terms (no interactions).

Model	Outcome	n	F p-value (HC3)	Predictor	R²	Adj. R²	Beta	Std. Error (HC3)	t-value	p-value	Partial R²	AIC
Asociality ~ covariates + NLR	Asociality	43	< 0.001	(Intercept)	0.37	0.24	6.07	2.52	2.41	0.021	-	212.10
				Sex			0.31	1.05	0.29	0.771	-	
				Age			0.04	0.04	1.09	0.281	0.04	
				BMI			-0.11	0.06	-1.73	0.093	0.12	
				Smoking			-0.01	0.05	-0.06	0.954	0.00	
				CPZ			-0.01	0.01	-1.33	0.191	0.07	
				Clozapine			1.82	1.07	1.70	0.097	-	
				NLR			0.77	0.29	2.67	0.011	0.16	
Emotional Withdrawal ~ covariates + NLR	N2	43	0.015	(Intercept)	0.33	0.20	2.91	1.34	2.17	0.037	-	157.68
				Sex			0.05	0.51	0.09	0.927	-	
				Age			0.03	0.02	1.46	0.153	0.07	
				BMI			-0.06	0.02	-2.35	0.024	0.11	
				Smoking			0.01	0.02	0.32	0.747	0.00	
				CPZ			-0.00	0.00	-0.41	0.681	0.01	
				Clozapine			1.11	0.50	2.23	0.032	-	
				NLR			0.36	0.16	2.25	0.031	0.13	
MAP ~ covariates + NLR	MAP	43	0.005	(Intercept)	0.32	0.19	20.39	7.85	2.60	0.014	-	311.88
				Sex: Male			0.26	3.44	0.08	0.941	-	
				Age			0.20	0.12	1.62	0.114	0.08	
				BMI			-0.32	0.18	-1.79	0.082	0.10	
				Smoking			-0.06	0.13	-0.48	0.637	0.01	
				CPZ			-0.01	0.01	-1.88	0.069	0.10	
				Clozapine			5.18	2.98	1.74	0.091	-	
				NLR			1.82	1.03	1.76	0.087	0.10	
Avolition ~ covariates + CRP	Avolition	43	0.495	(Intercept)	0.15	-0.02	6.94	2.59	2.68	0.011	-	217.47
				Sex: Male			-0.16	1.09	-0.14	0.886	-	
				Age			0.04	0.04	1.02	0.316	0.03	
				BMI			-0.07	0.08	-0.94	0.353	0.04	
				Smoking			-0.03	0.04	-0.70	0.489	0.01	
				CPZ			-0.00	0.00	-0.89	0.381	0.03	
				Clozapine			0.62	1.03	0.60	0.553	-	
				CRP			0.93	0.50	1.86	0.072	0.09	

Sensitivity analyses restricted to non-clozapine patients showed attenuation but persistence of the NLR-symptom associations across Asociality, N2, and MAP (ps = .065-.13). In models further adjusted for PANSS Positive and General Psychopathology scores, the Asociality model that included NLR remained the best-fitting specification (adjusted R² = .26, lowest AIC), while the NLR model for Emotional Withdrawal performed similarly to the covariate + PANSS-only model. This pattern suggests that the NLR-Asociality and NLR-N2 associations are not fully accounted for by overall symptom severity.

## Discussion

In this study, we hypothesized that integrating contemporary and standard measures of negative symptoms (BNSS and PANSS) with relevant clinical factors and routinely available inflammatory markers (CRP, NLR, and C4) would help identify specific symptom-clinical-biomarker patterns. Overall, our findings indicate that (1) BMI was inversely associated with negative symptoms-overweight participants showed lower symptom severity-and negative symptoms were higher among patients treated with clozapine; (2) in stratified analyses, Avolition was positively associated with CRP in non-overweight patients, whereas Asociality and the MAP factor were positively associated with NLR in clozapine-treated patients; and (3) in covariate-adjusted, AIC-selected regression models, the clearest biomarker signal emerged within the motivational domain. Across outcomes, models including NLR were consistently selected for Asociality and Emotional Withdrawal, with higher NLR linked to greater severity in both dimensions. In contrast, CRP showed only a modest, trend-level association with Avolition and did not contribute to other motivational outcomes. C4 did not enter any final multivariable model, although exploratory analyses suggested a sex-related divergence that warrants further investigation.

We observed a moderate and statistically significant inverse association between BMI and negative symptoms, indicating that lower BMI was associated with more severe negative symptoms. Consistent with this dimensional finding, participants with higher body mass tended to exhibit less pronounced negative symptomatology. Previous studies have likewise reported less severe negative symptoms among individuals with higher BMI [19,20]. This finding has been interpreted in two, not mutually exclusive, ways: first, that antipsychotic-related weight gain may accompany therapeutic improvement in negative symptoms; and second, that more severe negative symptoms (particularly anhedonia and reduced motivation) may lead to diminished reward processing, lower appetite, and consequently reduced BMI [19]. In our sample, however, greater negative symptom severity was also consistently observed among patients treated with clozapine, suggesting that clozapine use may predominantly index a more severe, treatment-resistant clinical subgroup characterized by prominent negative symptoms rather than symptomatic improvement in this domain [21].

Because BMI was positively associated with CRP (ρ(43) = .67) and negatively associated with negative symptoms, it likely confounded crude CRP-symptom associations. In line with this interpretation, among non-overweight participants CRP correlated positively with Avolition (ρ(17) = .49). In the AIC-selected multivariable model for Avolition-including sex, age, BMI, smoking, CPZ dose, clozapine, and CRP-overall model fit was modest (adjusted R² = .03), but higher CRP values remained associated with greater Avolition severity at a trend level (β = 0.93, p = .072; partial R² = .09). This pattern is directionally consistent with proposals linking low-grade inflammation, indexed by CRP, to motivational deficits via altered reward circuitry [22], while also highlighting that, in our data, CRP accounted for only a small proportion of unique variance once relevant covariates were controlled.

NLR showed a more consistent relationship with motivational negative symptoms. Among clozapine-treated patients, NLR displayed the strongest unadjusted correlations with Asociality and MAP (ρ(11) = .59-.66), and in the full sample, the AIC-selected models for Asociality and Emotional Withdrawal (PANSS N2) both retained NLR as an explanatory term. Higher NLR was associated with greater Asociality (β = 0.77, p = .011; partial R² = .16) and more severe Emotional Withdrawal (β = 0.36, p = .031; partial R² = .13). For MAP, the same covariate + NLR model was selected, but the NLR effect was only at a trend level (β = 1.82, p = .087; partial R²= .10), with additional trends for BMI and clozapine dose. Conceptually, NLR indexes the balance between innate and adaptive immune activity [23], and several recent studies have linked higher NLR to motivational negative symptoms [24,25]. In our sensitivity analyses restricted to non-clozapine patients and in models additionally adjusted for PANSS Positive and General Psychopathology scores, the direction and approximate magnitude of the NLR-Asociality and NLR-N2 associations were preserved, though p values shifted into the trend range, consistent with limited power and the exploratory nature of the study. Conversely, the stronger NLR-motivation signals observed among clozapine users may reflect the greater illness severity typical of this treatment-resistant subgroup [26] and clozapine’s known immunomodulatory effects (including transient NLR elevations and broader inflammatory changes) reported in prior work [27].

Although altered C4 levels have been reported in schizophrenia [28], their relationship with symptom dimensions remains uncertain. In our sample, C4 showed no meaningful associations with negative symptom dimensions in multivariable models. It did not improve model fit for any outcome, suggesting that peripheral C4 levels are not strongly relevant to motivational or expressive deficits in this inpatient cohort. Exploratory analyses did reveal a moderate inverse association between C4 and positive symptoms, consistent with evidence that complement activity may relate more strongly to psychosis liability or acute symptom expression [29]. Notably, we also observed a sex-divergent pattern, with C4 correlating positively with negative symptoms in men but negatively in women. Although these findings are underpowered and should be interpreted with caution, they parallel emerging reports of sex-specific complement regulation in schizophrenia [30] and raise the possibility of sex-dependent immunobiological pathways. Future studies with larger, longitudinal cohorts are needed to determine whether these preliminary patterns reflect stable mechanistic differences or sample-specific variation.

This study has several limitations. First, the modest sample size likely limited statistical power, and a larger cohort might have yielded more stable estimates, particularly for effects that approached statistical significance. Second, the sample consisted exclusively of hospitalized patients, a clinical context in which positive symptoms are often prominent, and negative symptoms may partially reflect secondary phenomena, rather than primary, enduring deficits. Importantly, the goal of the present study was not to differentiate primary from secondary negative symptoms, but rather to characterize the severity and structure of negative symptoms using a modern assessment instrument and to examine their associations with relevant clinical and biological dimensions. Third, the cross-sectional design precludes causal inference and limits the distinction between trait-related and state-dependent biological alterations, particularly for markers such as C4 that may fluctuate with inflammatory activity. Fourth, the inclusion of clozapine-treated patients poses specific challenges, as clozapine is known to influence leukocyte counts and inflammatory indices such as NLR. Nonetheless, these patients were retained because they constitute a clinically important, treatment-resistant subgroup in whom negative symptoms are often pronounced. Finally, we did not apply formal correction for multiple testing, in part because of the small sample size and the structured, hypothesis-generating analytic strategy. Consequently, the risk of type I error is non-negligible, and findings-especially those near conventional significance thresholds-should be interpreted cautiously and require independent replication.

Strengths. To our knowledge, this study is the first investigation conducted in a Romanian sample of patients with schizophrenia to assess negative symptoms using the BNSS in conjunction with routinely available peripheral inflammatory biomarkers in hospital settings. This is particularly relevant in the Romanian context, where inpatient care remains a central component of psychiatric services. Second, the study adds to a still-limited and emerging body of literature linking inflammation to BNSS-assessed negative symptoms, while simultaneously integrating key clinical factors, such as BMI and treatment status, into a unified clinico-biological framework.

## Conclusions

This study offers preliminary, hypothesis-generating evidence on the clinical and inflammatory correlates of negative symptoms in schizophrenia. First, lower BMI and clozapine treatment were both associated with greater severity of negative symptoms across BNSS and PANSS measures. Second, NLR emerged as the most consistent independent correlate of motivational negative symptoms, demonstrating associations across both BNSS (Asociality) and PANSS (Emotional Withdrawal) domains. CRP showed only weaker, less stable associations (primarily limited to Avolition), while C4 did not demonstrate any meaningful independent relationship with negative symptom dimensions. Third, BMI and clozapine status influenced (but did not fully explain) these relationships, suggesting that clinical and inflammatory factors interact in shaping motivational deficits. Given the modest effect sizes and limited generalizability, these findings warrant replication in larger, longitudinal samples.

## Figures and Tables

**Figure 3:**
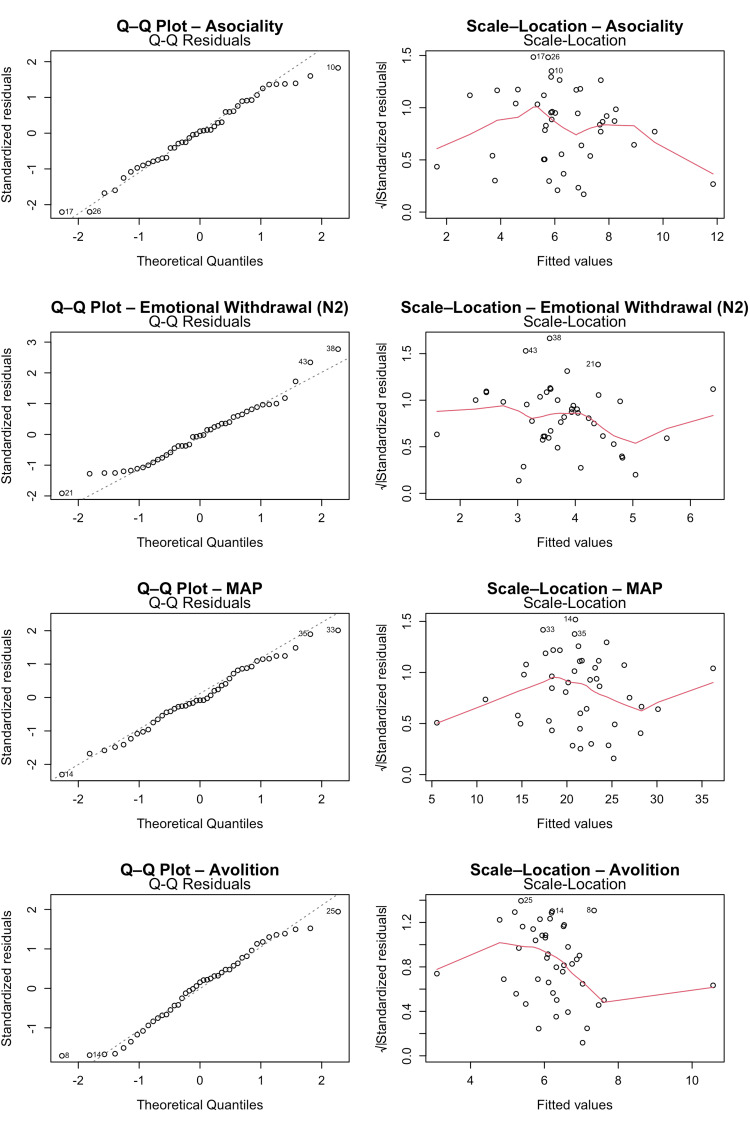
Residual Diagnostics for Final AIC-Selected Regression Models (Asociality, Emotional Withdrawal (N2), Motivation & Pleasure (MAP), and Avolition). Negative symptoms were assessed using two instruments: PANSS [17] item N2; and the BNSS [7], which provided scores for Asociality, Avolition, and MAP (Motivation and Pleasure) factor. Each row displays diagnostic plots for one outcome: a Q–Q plot of standardized residuals (left) assessing normality, and a Scale–Location plot (right) evaluating homoscedasticity. Across all four models, residuals aligned closely with the theoretical normal distribution, with only mild tail deviations expected in small samples. Scale–Location plots showed no systematic trends in residual spread, indicating approximate homoscedasticity. Together, these diagnostics support the suitability of linear regression for these summed-symptom outcomes. All models were estimated using HC3 heteroskedasticity-robust standard errors, further mitigating sensitivity to minor assumption violations. AIC = Akaike Information Criterion; BNSS = Brief Negative Symptom Scale; PANSS = Positive and Negative Syndrome Scale.

## Appendices

 Appendix 1

**Table 5:** Correlations Between Symptoms and Biomarkers. Negative symptoms were assessed using two instruments: PANSS [17] items N1, N2, N3, N4, N6, and the PANSS Negative Subscale; and the BNSS [7], which provided scores for total severity, Anhedonia, Asociality, Avolition, Blunted Affect, Alogia, as well as the MAP (Motivation and Pleasure) and EXP (Expressive) factor. Values show Spearman or Pearson r. Bold = *p < .05. CRP = C-reactive protein; C4 = Complement C4; NLR = Neutrophil-to-lymphocyte ratio.

Symptom	CRP	C4	NLR
Blunted Affect (N1)	–0.13	–0.01	0.02
Emotional Withdrawal (N2)	–0.08	–0.16	0.26
Poor Rapport (N3)	–0.30*	–0.08	–0.12
Passive Social Withdrawal (N4)	–0.04	–0.14	0.16
Lack of Spontaneity (N6)	–0.20	0.04	–0.02
PANSS Negative	–0.13	–0.11	0.09
BNSS Total	–0.14	–0.00	0.15
Anhedonia	–0.15	–0.01	0.19
Asociality	–0.15	–0.01	0.23
Avolition	0.16	–0.11	0.24
Blunted Affect	–0.09	–0.12	0.11
Alogia	–0.32*	0.02	0.06
MAP	–0.07	–0.01	0.23
EXP	–0.19	–0.04	0.08
Lack of Normal Distress	–0.12	–0.10	–0.07
PANSS Positive	0.07	–0.36*	–0.02
PANSS General	–0.01	–0.27	0.35*

Appendix 2 
